# ASO Author Reflections: sCD40 as a Preoperative Predictor in Pancreatic Cancer Surgery

**DOI:** 10.1245/s10434-026-19716-9

**Published:** 2026-05-02

**Authors:** Loreen Natusch Bufe, Adrian M. Seifert, Lena Seifert

**Affiliations:** 1 Department of Visceral, Thoracic, and Vascular Surgery, University Hospital Carl Gustav Carus, Dresden, Germany; 2 Technische Universität Dresden Medizinische Fakultät Carl Gustav Carus, Dresden, Germany; 3 National Center for Tumor Diseases Dresden, Dresden, Germany; 4 Partner Site Dresden, German Cancer Consortium, Heidelberg, Germany

## Past

Pancreas-specific complications following pancreatic cancer surgery remain a major clinical challenge, contributing significantly to postoperative morbidity and mortality.^1^ In particular, postoperative pancreatic fistula (POPF) and postpancreatectomy hemorrhage (PPH) ^2-4^ can delay or even preclude the initiation of adjuvant chemotherapy. Reliable preoperative biomarkers to predict these complications are currently missing.

## Present

In this retrospective study, preoperative serum samples from pancreatic ductal adenocarcinoma (PDAC) patients, undergoing pylorus-preserving pancreatoduodenectomy or a Whipple procedure, were included.^5^ Our study demonstrates that reduced preoperative sCD40 serum levels are associated with an increased risk of postoperative pancreas-specific complications, including POPF and PPH (*P* = 0.025 and *P* = 0.008), after pancreaticoduodenectomy. Receiver operating characteristic (ROC) analysis revealed that sCD40 alone has modest discriminatory ability (AUC = 0.660). However, combining sCD40 with leukocyte count and body mass index (BMI) improved predictive performance (AUC = 0.705 for POPF and 0.752 for PPH), suggesting added value in multimodal risk assessment.^5^

## Future

These findings identify sCD40 as a promising preoperative biomarker for predicting pancreas-specific complications. Accordingly, sCD40 may offer additional value for preoperative risk stratification and represents a promising candidate for inclusion in future predictive models to identify patients at high risk for POPF or PPH. However, prospective validation in larger cohorts is needed to confirm these findings and to establish sCD40 as a clinically applicable biomarker, potentially enabling individualized perioperative management and improved patient outcomes.
